# Opioids alter paw placement during walking, confounding assessment of analgesic efficacy in a postsurgical pain model in mice

**DOI:** 10.1097/PR9.0000000000001035

**Published:** 2022-08-25

**Authors:** Victoria E. Brings, Maria A. Payne, Robert W. Gereau

**Affiliations:** a Washington University Pain Center and Department of Anesthesiology, Washington University School of Medicine, St. Louis, MO, USA; Departments of b Neuroscience and; c Biomedical Engineering, Washington University, St. Louis, MO, USA

**Keywords:** Opioid, Oxycodone, Gait, Paw incision, Postsurgical injury, Mouse behavior

## Abstract

**Introduction:**

Hind paw–directed assays are commonly used to study the analgesic effects of opioids in mice. However, opioid-induced hyperlocomotion can obscure results of such assays.

**Objectives:**

We aimed to overcome this potential confound by using gait analysis to observe hind paw usage during walking in mice.

**Methods:**

We measured changes in the paw print area after induction of postsurgical pain (using the paw incision model) and treatment with oxycodone.

**Results:**

Paw incision surgery reduced the paw print area of the injured hind paw as mice avoided placing the incised section of the paw on the floor. Surprisingly, oxycodone caused a tiptoe-like gait in mice, reducing the paw print area of both hind paws. Further investigation of this opioid-induced phenotype revealed that analgesic doses of oxycodone or morphine dose-dependently reduced the hind paw print area in uninjured mice. The gait changes were not dependent on opioid-induced increases in the locomotor activity; speed and paw print area had no correlation in opioid-treated mice, and other analgesic compounds that alter locomotor activity did not affect the paw print area.

**Conclusion:**

Unfortunately, the opioid-induced “tiptoe” gait phenotype prevented gait analysis from being a viable metric for demonstrating opioid analgesia in injured mice. However, this work reveals an important, previously uncharacterized effect of treatment with analgesic doses of opioids on paw placement. Our characterization of how opioids affect gait has important implications for the use of mice to study opioid pharmacology and suggests that scientists should use caution when using hind paw–directed nociceptive assays to test opioid analgesia in mice.

## 1. Introduction

Mice are widely used to study the behavioral effects of opioids, including analgesia, dependence, and respiratory depression.^8,13,29,32^ Antinociceptive properties of mu-opioid agonists are often tested in mice using reflexive hot-plate or tail-flick assays.^13,32,36^ To study opioid analgesia in tissue or nerve injury models in mice, injury-induced sensitization and its reversal with opioids are assessed by measuring sensitivity to stimuli directed to the affected body region, which is often the hind paw.^12^ Most commonly used hind paw–directed assays entail the quantification of a withdrawal response to the application of noxious stimuli to the hind paw.^5,12^ These assays reflect evoked responses to an exogenous stimulus and do not reflect naturalistic pain behaviors.^12,33^ They also measure reflexive withdrawal responses, which may not accurately represent pain or the effects of analgesia.^15,19^ In addition to having potentially flawed face validity, hind paw–directed evoked pain assays are particularly challenging to perform on opioid-treated mice because of increases in the locomotor activity induced by mu-opioid agonists.^10,35,36^

Because of the shortcomings of evoked withdrawal tests, alternative approaches are needed to assess opioid analgesia in mice. We and others have used a variety of nonreflexive behavioral assays to assess pain sensitivity and analgesia in mice, such as voluntary wheel running, home cage lid hanging, and gait analysis.^22,31,33,38,42^ The Noldus CatWalk XT gait analysis system was originally designed to quantify deficits in motor function seen in models of spinal cord injury.^16,17^ More recently, others have used this system to demonstrate the effects of injury and analgesic treatment on gait in rats and mice.^1,22,31,38^ To achieve our goal of measuring behavior affected by postsurgical injury and reversed by opioids in mice, we used the CatWalk gait analysis system to observe hind paw usage during voluntary walking behavior. Gait analysis provides a means of assessing hind paw usage with a nonreflexive behavioral assay that would be technically feasible with a hyperactive mouse.

Using the CatWalk system, we measured changes in the paw print area after induction of postsurgical pain with the paw incision injury model and treatment with oxycodone in mice. We hypothesized that paw incision injury would produce alterations in paw placement during walking that could be reversed with oxycodone. We found that paw incision injury reduces the print area of the injured paw. Surprisingly, however, analgesic doses of oxycodone induced a “tiptoe” phenotype that dose dependently reduced the print area of all paws. The reduction in the paw print area was independent of walking speed and was specific to opioids (oxycodone and morphine), as mice treated with the nonopioid analgesics fenobam and Δ^9^-tetrahydrocannibinol did not show altered paw print area. Overall, this work demonstrates the effect of opioid treatment on paw usage and has implications for the use of hind paw–directed sensory assays in opioid-treated mice.

## 2. Methods

### 2.1. Animals

All procedures used in this study were approved by the Institutional Animal Care and Use Committee at Washington University in St. Louis School of Medicine. C57BL/6 mice bred in-house were given food and water ad libitum in a facility with a 12-h light:dark cycle. Behavior was tested during the light phase at 8 to 12 weeks of age. Male and female mice were tested separately. Animals were randomly assigned to each condition. Different test groups (injury and drug treatment) had equal average weights. Cages had mixes of animals from different test groups, group-housed with 3 to 5 mice per cage. Experimenters were blinded to drug treatment or surgical condition until data were analyzed.

### 2.2. Drugs

Oxycodone (USP, Rockville, MD) was dissolved in saline (0.9% NaCl; Hospira, Lake Forest, IL), and morphine (10 mg/mL; Hikma, Eatontown, NJ) was diluted in saline; each was delivered s.c. in a volume of 5 mL/kg body weight. Δ^9^-tetrahydrocannabinol (THC; 200 mg/mL; National Institute on Drug Abuse Drug Supply Program, Bethesda, MD) was diluted with 95% ethanol followed by a mix of Kolliphor EL (Sigma-Aldrich, St. Louis, MO) and saline to achieve a final vehicle of 5% 95% ethanol, 5% Kolliphor EL, and 90% 0.9% saline, delivered s.c. in a volume of 5 mL/kg. Fenobam (SCYNEXIS, Durham, NC) was dissolved in a vehicle of 100% DMSO (Sigma-Aldrich), delivered i.p. in a volume of 20 μL. For all drugs, testing was performed within 30 minutes (30–60 minutes postdelivery of oxycodone, morphine, and Δ9-THC; 5–35 minutes postdelivery of fenobam). Doses were chosen based on previous reports that analgesia and/or antinociception can be achieved at the indicated doses for oxycodone,^2,14,26,30,39,40^ morphine,^26,30^ fenobam,^25,27,28^ and Δ^9^-THC.^3,9,23^ Mice were returned to home cage after injection and were kept there until testing.

### 2.3. Paw incision surgery

**Figure 1.**
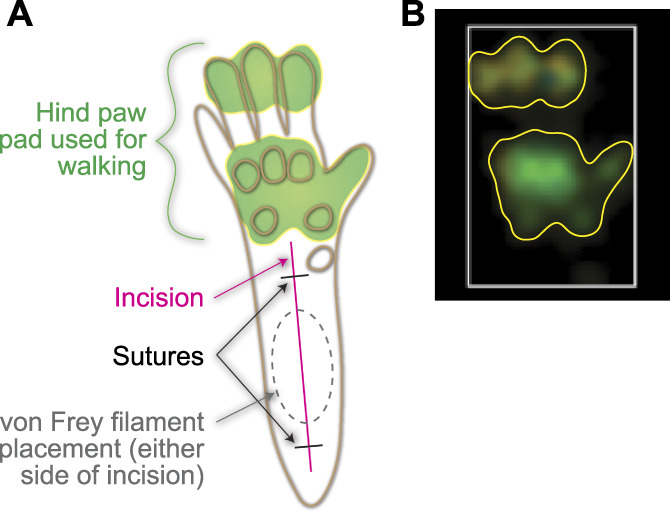
Diagram of the hind paw showing locations used for sensory testing and incision injury. (A) An outline of a mouse hind paw showing circles that correspond to raised sections of the foot pad (brown). The approximate size and location of the incision (magenta) and the 2 sutures that close the wound (black) are also shown, along with the area that the von Frey filament was placed during sensory testing (gray dashed circle). The area of the hind paw that makes contact with the ground is indicated (green). (B) A paw print image produced by the Noldus CatWalk software (white box surrounding the paw print added by the software) from a mouse walking without injury or drug treatment. Yellow outlines added to A and B indicate the outline of the paw print captured by the software and the corresponding hind paw anatomy that generated the print.

Paw incision surgery was performed on the right hind paw (Fig. 1A), following previous published procedures.^11,41^ While under isoflurane anesthesia, the surface of the right hind paw was sterilized and a 5-mm incision was made through the skin starting 2 mm from the heel using a type 11 scalpel blade (McKesson, Richmond, VA). The flexor digitorum brevis muscle was pulled up to isolate, and an approximately 3-mm vertical incision was made through the muscle. The skin was closed with 2 6-0 silk sutures (Ethicon, Guaynabo, Puerto Rico). Antibiotic ointment (McKesson) was applied. For the sham surgery, mice were anesthetized, the right hind paw was sterilized, and sutures were applied, but no incisions were made.

### 2.4. Electronic von Frey

Mechanical allodynia was measured using an electronic von Frey anesthesiometer (IITC Life Science, Woodland Hills, CA).^34^ Mice were placed on an elevated stainless steel mesh platform and isolated in individual acrylic containers. Mice were acclimated for at least 1 day and were habituated for 1 hour before testing. Alternating hind paws, 5 measurements were taken per paw, with at least 1 minute between measurements for a single paw. On the day of testing postsurgery, approximately 15 minutes after acquiring baseline data, the animals were injected with the test compound and tested again for postdrug responses 30 minutes later. The blunt tip of the anesthesiometer was applied to the hind paw approximately 4 to 5 mm from the heel (corresponding to the space between the 2 sutures on the hind paw in sham and injured animals, Fig. 1A), and the amount of force applied before the animal withdrew their hind paw was recorded. Of the 5 measurements taken per paw, the highest and lowest measurements were excluded, and the remaining 3 measurements were averaged.

### 2.5. CatWalk gait analysis

The Noldus CatWalk XT gait analysis system (Noldus Information Technology, Leesburg, VA) was used to analyze the paw prints of mice.^1,22,31,33,38^ In a dark room, mice walked across an elevated glass platform with a camera positioned underneath, facing the underside of the animal. Light illuminated the section of the paws that applied weight on the glass (Fig. 1A). Paw prints were imaged by the camera and measured using the Noldus CatWalk XT 10 software (Fig. 1B). The paw print area refers to the total area of the glass in contact with the paw, measured at the point in the step when the area of contact with the glass is maximal for that paw print. Analgesic studies in incision animals were performed in mice that had a relative paw print area in the ipsilateral paw that was no greater than 80% than of the contralateral paw; 10 of 46 mice were thus excluded based on this criterion.

### 2.6. Imaging mouse walking behavior

Mice walked across an elevated glass platform and were imaged with a Google Pixel 4 (Google, Mountain View, CA).

### 2.7. Statistics

Data were analyzed using GraphPad Prism 9.3.0 (GraphPad Software, San Diego, CA). Data were analyzed with one-way or two-way analysis of variance (ANOVA) tests, followed by Dunnett or Tukey multiple comparisons tests, respectively. When 2 groups were being compared, *t* tests were used (paired or unpaired, as indicated below). A simple linear regression was used to test the correlation between the paw print area and speed. Statistical values and tests used are described in the text below. Significant main effects are reported in the text, and significant post hoc test results are indicated in the figures.

## 3. Results

### 3.1. Oxycodone reverses incision-induced mechanical allodynia but not reduced paw print area of the injured paw

**Figure 2.**
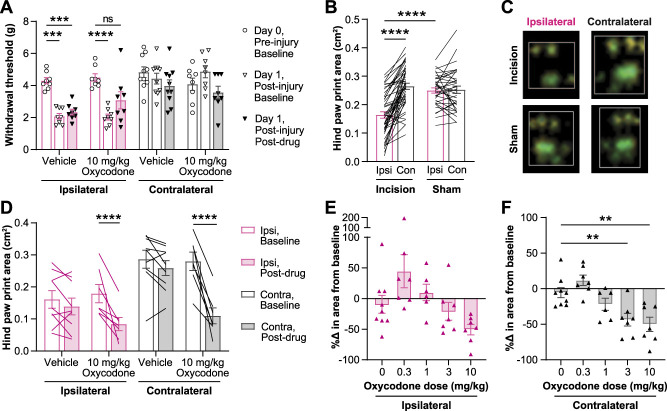
Oxycodone reverses mechanical allodynia but not paw print changes after paw incision surgery. (A) Withdrawal threshold was measured in contralateral and ipsilateral hind paws before paw incision surgery, 1 day after surgery, and 30 minutes after treatment with 10 mg/kg of oxycodone or vehicle on day 1 after surgery. (B) Paw incision surgery reduced the paw print area of the injured (ipsilateral) hind paw, relative to the animals' contralateral paw or to sham animals' ipsilateral paws. (C) View of the hind paw print image collected by the CatWalk system when the paw makes maximum contact with the glass in a paw incision injured mouse. (D) The paw print area was measured in both hind paws twice on the day after surgery: before (baseline) and after (postdrug) treatment with 10 mg/kg of oxycodone or vehicle. (E–F) On day 1 after paw incision surgery, the percent change in the paw print area from baseline to postdrug (30 minutes after treatment with indicated oxycodone doses) in the ipsilateral (E) and contralateral (F) hind paws. All oxycodone administration was s.c., 30 minutes before the postdrug test. Mean ± SEM. Showing results of posttests following 2-way ANOVAs for A (*P* < 0.0001), D (*P* < 0.0001), and F (*P* < 0.0001); **P < 0.01, ***P < 0.001, ****P < 0.0001. (B) Unpaired *t* test of sham vs incision ipsilateral; paired *t* test of incision contralateral vs ipsilateral; ****P < 0.0001. ANOVA, analysis of variance; Con, contralateral; Ipsi, ipsilateral.

Consistent with prior reports, we find that paw incision produces robust sensitization to mechanical stimuli in mice as assessed using von Frey 1 day after injury (Fig. 2A). After treatment with oxycodone (10 mg/kg, s.c.) on day 1 postsurgery, the withdrawal thresholds were no longer significantly different from preinjury baseline values (F_6, 60_ = 7.097, *P* < 0.0001, 2-way ANOVA interaction effect).

Paw incision alters paw placement by inducing paw guarding behavior in rats and mice.^4,41^ Therefore, we hypothesized that the CatWalk gait analysis system, which evaluates paw placement during walking, would detect altered paw usage in injured mice. We performed paw incision surgery and analyzed the paw print area during walking using the CatWalk system 1 day postsurgery. Paw incision surgery reduced the paw print area of the injured (ipsilateral) hind paw, relative to the animal's contralateral paw (*P* < 0.0001, paired *t* test) or to sham animals' ipsilateral paws (*P* < 0.0001, unpaired *t* test; Fig. 2B). Viewing images of the paw prints shows that the reduction in the paw print area is due to an avoidance of placing weight on the proximal section of the paw, closer to the site of the incision (Fig. 2C).

Next, we assessed if oxycodone could reverse the incision-induced reduction in the paw print area (Fig. 2D). As in Figure 2B, the paw print area was reduced in injured hind paws relative to contralateral paws 1 day postsurgery. We hypothesized that oxycodone would increase the contact area of the injured paws. Surprisingly, however, oxycodone (10 mg/kg) decreased the paw print area of both uninjured and injured hind paws (F_4, 31_ = 10.64, *P* < 0.0001, 2-way ANOVA interaction effect)*.* In other injury models, different doses of oxycodone were required to reverse sensitization phenotypes measured with different behavior tests.^26,40^ Therefore, we sought to determine if a lower dose of oxycodone would reverse the impaired paw placement in the incised paws without affecting the contralateral paws (Fig. 2E, F). No dose of oxycodone significantly altered the paw print area of the injured paw relative to the vehicle (ie, 0 mg/kg of oxycodone) treatment (Fig. 2E); however, 3 and 10 mg/kg of oxycodone significantly decreased the area of the contralateral paw prints relative to the vehicle (F_4, 31_ = 9.589, *P* < 0.0001, 1-way ANOVA; Fig. 2F).

### 3.2. Opioids dose-dependently decrease the paw print area during walking

**Figure 3.**
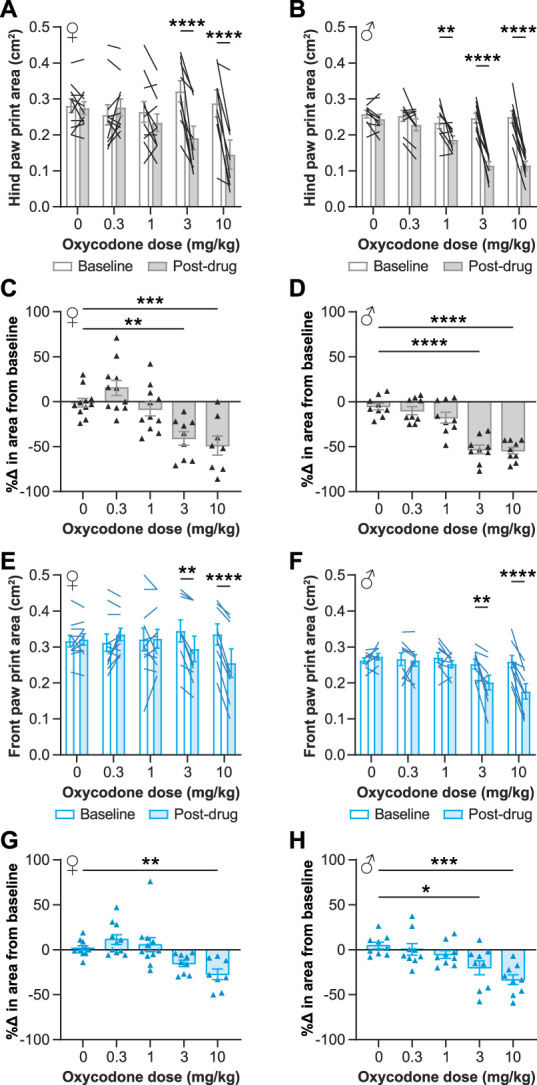
Analgesic doses of oxycodone reduce the paw print area in female and male mice. Analgesic doses of oxycodone reduced the hind paw (A–D) and front paw (E–H) print areas in uninjured female (A, C, E, G) and male (B, D, F, H) mice. (A, B, E, F) The area of the paws at the point of maximum paw contact before (baseline) and after (postdrug) oxycodone administration. (C, D, G, H) The percent change between baseline and postdrug for each animal. Mean ± SEM. Showing results of posttests following 2-way ANOVAs for A (*P* < 0.0001), B (*P* < 0.0001), E (*P* < 0.0001), and F (*P* < 0.0001) and 1-way ANOVAs for C (*P* < 0.0001), D (*P* < 0.0001), G (*P* < 0.0001), and H (*P* = 0.0001); *P < 0.05, **P < 0.01, ***P < 0.001, ****P < 0.0001. ANOVA, analysis of variance.

Based on the finding of oxycodone's effect on the contralateral paw print area, we further investigated the effect of oxycodone on paw placement in uninjured mice. Using a range of analgesic doses of oxycodone,^2,14,26,30,39,40^ we assessed paw placement during walking before and after oxycodone treatment, as in Figure 2D. We found that oxycodone caused dose-dependent reductions in the print area of the hind paws in both female (F_4, 45_ = 12.48, *P* < 0.0001, 2-way ANOVA interaction effect; Fig. 3A) and male (F_4, 40_ = 19.86, *P* < 0.0001, 2-way ANOVA interaction effect; Fig. 3B) mice, and the effect of oxycodone was greater than that of vehicle in females (F_4, 45_ = 11.67, *P* < 0.0001, one-way ANOVA; Fig. 3C) and males (F_4, 40_ = 26.65, *P* < 0.0001, one-way ANOVA; Fig. 3D), as well. In females, the hind paw print area was reduced after 3 or 10 mg/kg oxycodone treatment (Fig. 3A), and in males, the hind paw print area was reduced after 1, 3, or 10 mg/kg oxycodone treatment (Fig. 3B), relative to baseline print areas. The front paw print area was also significantly reduced by oxycodone in a dose-dependent fashion in both females (F_4, 45_ = 11.27, *P* < 0.0001, 2-way ANOVA interaction effect; Fig. 3E) and males (F_4, 40_ = 7.980, *P* < 0.0001, 2-way ANOVA interaction effect; Fig. 3F). Significant reductions relative to baseline were seen after 3 and 10 mg/kg oxycodone treatment in both female and male front paw print areas, and, as with the hind paws, the change in the paw print area induced by oxycodone was significantly greater than that induced by vehicle in females (F_4, 45_ = 7.771, *P* < 0.0001, one-way ANOVA; Fig. 3G) and males (F_4, 40_ = 7.539, *P* = 0.0001, one-way ANOVA; Fig. 3H).

**Figure 4.**
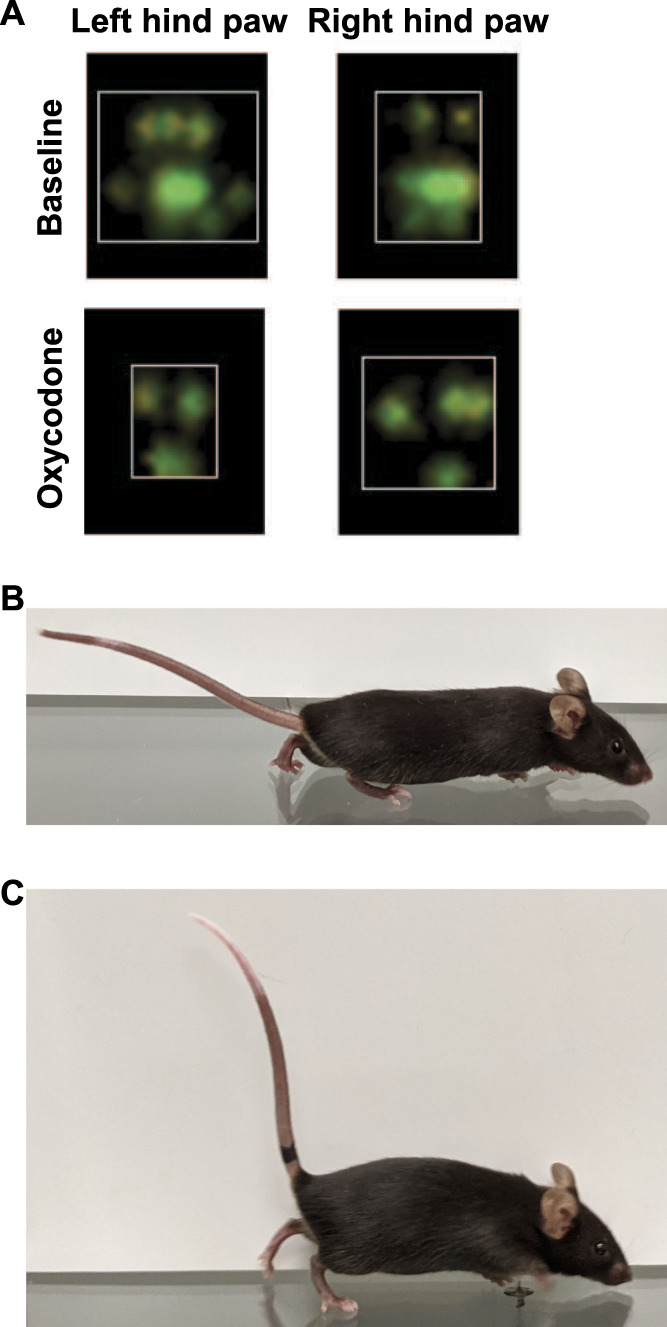
Analgesic doses of oxycodone reduce the paw print area by inducing tiptoe walking. (A) Paw prints were imaged by the Noldus CatWalk software. White boxes show edges of the paw print. Mice were photographed while walking on an elevated glass platform 30 minutes after treatment with vehicle (B) or 10 mg/kg of oxycodone (C). The oxycodone-treated mouse shows incomplete placement of the hind paw on the glass, elevated body position relative to the platform, and a rigid Straub tail.

To assess how oxycodone alters paw placement to reduce the paw print area, we examined paw print images and images of animals walking after oxycodone treatment. Images of the paw prints when the maximum contact is made with the glass showed that mice favored the distal parts (toward the toes) of their feet after treatment with 10 mg/kg of oxycodone relative to their walking behavior at baseline (Fig. 4A). While observing mice at the moment when the hind paw first lands during a step, we saw that vehicle-treated mice (Fig. 4B) placed their full hind paw pad down on the glass and that 10 mg/kg oxycodone-treated mice (Fig. 4C) avoided placing the more proximal section of their paws (ie, their heels) down on the glass. Oxycodone-treated mice also showed rigid, vertically pointed tails and, by adopting a tip-toe–like posture while walking, were more elevated from the platform. The avoidance of heel placement seen after oxycodone treatment could thus produce a reduction in the paw print area.

**Figure 5.**
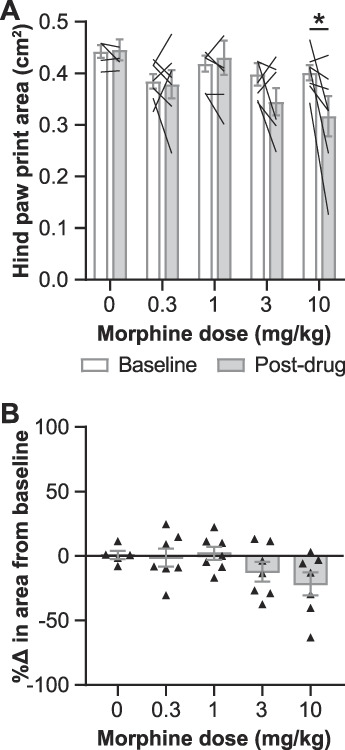
Analgesic doses of morphine reduce the hind paw print area during walking in female mice. (A) The area of the hind paws at the point of maximum paw contact before (baseline) and after (postdrug) morphine administration (s.c., 30 minutes before test). (B) The percent change in the paw print area between baseline and postdrug for each animal. Mean ± SEM. Showing results of posttests following a 2-way ANOVA for A (*P* = 0.0428); *P < 0.05. ANOVA, analysis of variance.

To determine if the effect on paw placement found with oxycodone could be seen with another analgesic mu-opioid receptor agonist, we examined paw placement on the CatWalk before and after morphine treatment. Morphine also dose-dependently decreased the paw print area. As with oxycodone, 10 mg/kg of morphine (s.c.) reduced the paw print area relative to baseline (F_4, 28_ = 2.840, *P* = 0.0428, 2-way ANOVA effect of drug; Fig. 5A, B).

### 3.3. Altered walking speed does not drive the reduced paw print area

**Figure 6.**
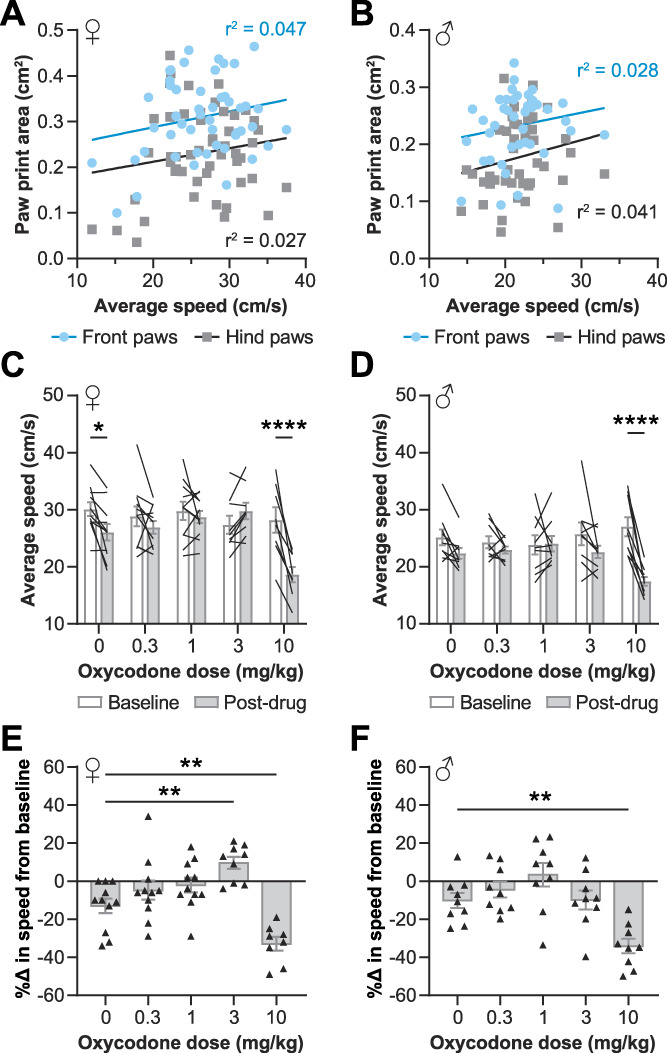
Oxycodone's effect on walking speed does not drive altered paw print area. Speed and front or hind paw print area had no correlation in oxycodone-treated female (A) or male (B) mice. The average speed during walking on the CatWalk platform before (baseline) and after (postdrug) oxycodone administration in females (C) and males (D). The percent change between baseline and postdrug for each female (E) and male (F) mouse. Showing results of linear regression for A and B and posttests following 1-way ANOVAs for C (*P* < 0.0001), D (*P* = 0.0003), E (*P* < 0.0001), and F (*P* < 0.0001); *P < 0.05, **P < 0.01, ****P < 0.0001. (C–F) Mean ± SEM. ANOVA, analysis of variance.

The oxycodone doses used here significantly increase the distance covered in an open field,^10^ and, thus, with time being a constant, average speed in an open field is increased. Therefore, we asked whether altered walking speed could cause the opioid-induced tiptoe like gait. We saw no correlation between the paw print area and the speed of the mice (Fig. 6A, B).

Nearly all vehicle-treated mice had slower average speeds on the CatWalk during their postdrug trials relative to their baseline trials in female mice (F_4, 45_ = 9.736, *P* < 0.0001, 2-way ANOVA interaction effect; Fig. 6C), and both female and male mice (F_4, 40_ = 6.671, *P* = 0.0003, 2-way ANOVA interaction effect; Fig. 6D) were significantly slower during their post-10 mg/kg of oxycodone trials relative to baseline, as well. Notably, the reduction in speed during postdrug trials was significantly greater in the mice treated with 10 mg/kg of oxycodone relative to the change in speed in mice treated with the vehicle in both females (F_4, 45_ = 12.88, *P* < 0.0001, one-way ANOVA; Fig. 6E) and males (F_4, 40_ = 8.954, *P* < 0.0001, one-way ANOVA; Fig. 6F). Interestingly, in female mice, 3 mg/kg of oxycodone increased walking speed relative to baseline (Fig. 6E). The hind paw print area was decreased at both 3 and 10 mg/kg of oxycodone in females (Fig. 3A, C), but speed was increased at 3 mg/kg and decreased at 10 mg/kg of oxycodone. In addition, the effects of oxycodone on the hind paw area in males (Fig. 3B, D), particularly the equivalent effects of 3 and 10 mg/kg on paw placement, did not match the effects of oxycodone on walking speed (Fig. 6F), as only 10 mg/kg of oxycodone affected walking speed in male mice. Together, these data support the conclusion that the change in speed caused by oxycodone treatment did not drive the change in paw placement.

**Figure 7.**
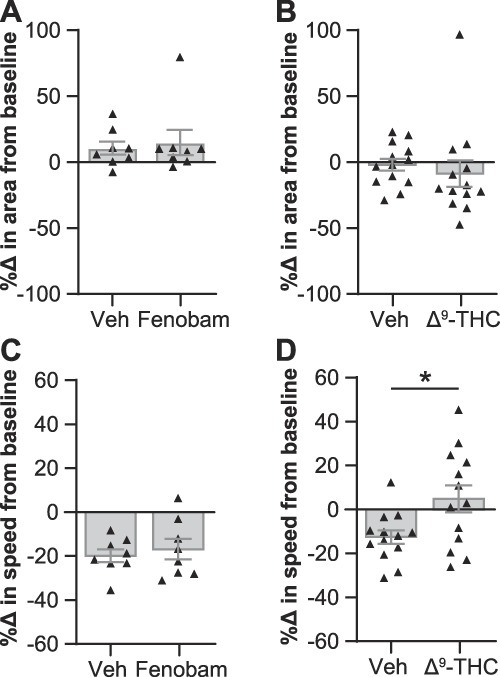
Nonopioid analgesics fenobam and Δ^9^-THC do not affect the paw print area. The percent change in the hind paw area (A and B) and average walking speed (C and D) between baseline and postdrug walking in mice treated with (A and C) fenobam (30 mg/kg, i.p., 5 minutes before test) and (B and D) Δ^9^-THC (3 mg/kg, s.c., 30 minutes before test). Mean ± SEM. Unpaired *t* test in D; *P < 0.05.

As an additional exploration into the effect of walking speed on paw placement, we tested the effects of 2 nonopioid analgesic drugs that differentially affect open field activity on the paw print area in female mice. Fenobam, an mGlu5 receptor negative allosteric modulator, reduces pain behaviors and increases open field activity.^27^ Δ^9^-THC, a cannabinoid type 1 and type 2 receptor agonist, provides antinociception and decreases open field activity.^3,9,23^ Neither fenobam (30 mg/kg, i.p.) nor Δ9-THC (3 mg/kg, s.c.) affected the paw print area differently from their vehicle-treated counterparts (Fig. 7A, B). Similarly to the oxycodone-treated mice, the effects on speed also did not match their respective effects on previously reported open field activity. Fenobam and vehicle treatment had equivalent effects on speed (Fig. 7C), and mice treated with Δ^9^-THC did not have the same reductions in walking speed as the vehicle-treated mice (*P* = 0.0192, unpaired *t* test; Fig. 7D).

## 4. Discussion

Overall, we found that paw incision surgery reduced contact of the incised section of the paw during walking on the CatWalk platform, resulting in a decrease in the paw print area of the injured hind paw. Treatment with analgesic doses of opioids induced a tiptoe like gait in injured and uninjured mice, causing reduced paw print area of both hind paws. Therefore, although the paw print area during walking may be used to as a phenotypic demonstration of postinjury sensitization, it is not one that can be reversed by opioids in mice, as opioid treatment presents a confounding phenotype.

Within the results showing that opioids induce tiptoe walking, notable differences were seen between some subgroups. At the same doses, morphine had smaller effects on the paw print area (Fig. 5A, B) than oxycodone did (Fig. 3A, C). Others have observed similar differences, as morphine has also been shown to be less analgesic in injured mice and has reduced antinociceptive efficacy in uninjured mice, relative to the same doses of oxycodone.^26,30^ In addition, females and males responded differently to the same oxycodone doses. In males, 1 mg/kg of oxycodone reduced the hind paw print area relative to baseline (Fig. 3B), whereas females were only affected at a dose as low as 3 mg/kg of oxycodone (Fig. 3A). The sex differences in the effects of oxycodone on the paw print area echo the literature demonstrating the enhanced sensitivity of male mice to the effects of opioids relative to female mice, including opioid-induced antinociception, tolerance, and locomotor activity.^7,10,24^

In addition to the effects of opioids on the paw print area, this work demonstrates an effect on walking speed that has not previously been quantified. Here, we show that male and female mice have a greater decrease in walking speed after treatment with 10 mg/kg of oxycodone than their vehicle-treated counterparts (Fig. 6C–F). This dose of oxycodone was previously reported to increase the total distance traveled in an open field in both sexes.^10^ An increase in distance traveled is typically interpreted as an increase in average speed for the whole experiment, as the time of an open field test is kept constant. However, mice spend large portions of the open field period immobile. Our results suggest that the 10 mg/kg oxycodone-induced increase in distance covered may instead be driven by an increase in the percent of time spent mobile, with the walking speed (the average speed during mobile periods, not during the whole course of the test) actually being lower than that of vehicle-treated mice. An increase in the percent of time spent walking would also lead to more distance covered, a result that is often interpreted as being due to faster walking. As researchers explore the side effects of opioids in mice, including locomotor sensitization, our results on the effects of oxycodone on speed may help people better evaluate their data. Similar differential effects were shown here for THC and fenobam; the average walking speed in CatWalk did not align with the average speed previously shown in an open field, as calculated using the total distance covered. CatWalk and open field experiments have different conditions (eg, floor material and lighting) that could contribute to different walking behaviors in each type of arena, but it would be interesting to parse out the effects on walking speed and time spent walking in an open field after treatment with these drugs as well.

One other mouse model that has shown tiptoe walking is the *twy* Yoshimura mouse, but the cause of tiptoe walking in this animal is a spinal injury arising from spinal ligament ossification and is unlikely to share a causal mechanism with acute opioid-induced tiptoe gait.^18^ Others have anecdotally noted the presence of tiptoe like walking induced by buprenorphine,^20,21^ but none have quantified this behavior, and the cause is unknown.

One known side effect of mu-opioid receptor agonist treatment that could cause altered paw placement is muscle rigidity. Rigid extension of the hind limbs resulting from subcutaneous morphine has been noted in mice^6^ and was quantified in hind limb electromyographic activity studies using rats treated with subcutaneous alfentanil.^37^ Muscle stiffness in the hind limbs could cause the back legs to be extended backward, preventing the heels of the hind paw from making contact with the walking surface, leading to a reduced hind paw print area. At higher oxycodone doses, this rigid extension may also occur in the fore limbs, leading to the reduced paw print area seen in the front paws. Similarly, muscle rigidity is seen in the tail (ie, Straub tail) in a dose-dependent fashion^6^ that correlates well with the dose-dependent effect of tiptoe walking (Fig. 4C), supporting the potential conclusion that muscle rigidity drives this effect.

An interesting effect on speed is seen in females treated with oxycodone; compared with their baseline speed, postdrug walking speed dose dependently increases with oxycodone treatment up to 3 mg/kg but is significantly decreased with 10 mg/kg of oxycodone. If muscle rigidity begins between 3 and 10 mg/kg in females, perhaps the hyperlocomotion effect of oxycodone begins to be outweighed by the muscle rigidity effect at doses above 3 mg/kg. In other words, oxycodone may dose-dependently increase the drive to be mobile (hence the increase in open field distance covered at 10 mg/kg^10^), but by 10 mg/kg, the muscle rigidity effect is strong enough to slow down the walking speed so that the mice are not walking faster, they are simply walking for longer.

Ideally, there would be a dose of oxycodone that would provide analgesia in incised animals without inducing tiptoe walking. Indeed, the effective dose of oxycodone can vary for different phenotypes of a single injury model; for example, in a mouse model of burn pain, the oxycodone dose required to reverse injury-induced CatWalk phenotypes was higher than the dose required to reverse injury-induced von Frey effects.^40^ However, in the paw incision mice tested here, the oxycodone doses that were low enough not to affect the paw print area in uninjured paws were not sufficient to reverse the paw print area phenotype in injured hind paws. The dose range at which analgesia is typically provided by oxycodone (1–20 mg/kg)^2,14,26,30,39,40^ overlaps with the dose range for tiptoe walking shown here (1–10 mg/kg; Fig. 3). Therefore, although the paw print area is a robust metric for demonstrating gait alterations induced by paw incision, this metric cannot be used to evaluate oxycodone's ability to provide analgesia in this injury model.

Additional assessments are needed to confirm that the incision-induced paw print area reductions are indeed a pain-related phenotype. Opioid treatment was shown to reverse paw usage impairments (guarding behavior) caused by paw incision^41^ and paw print area changes induced by other injury models^1,41^ in rats. Furthermore, the paw print area changes induced by neuropathic injury were reversed by gabapentin in mice.^22^ This suggests that altered paw placement is indeed a pain-related symptom that can be reversed by analgesics in rodents.

As a future direction, other metrics of paw usage could be analyzed to determine if they are altered by paw incision surgery and/or opioid treatment in mice. Whereas the CatWalk system measures 2-dimensional paw placement, the amount of weight placed on the paw can only be generally estimated based on the light intensity of the paw print. Oxycodone may not reverse the area of the injured paw that makes contact with the glass, but it may affect how much weight is being placed on that reduced foot print. Static or dynamic weight-bearing assays may be used as an alternative method to find a paw usage phenotype that is altered in an injured state and reversed by oxycodone. Having a better understanding of how paw usage varies with injury or oxycodone treatment, as well as how paw usage phenotypes manifest on test surfaces made of different materials, would also help determine if other paw-directed assays (eg, hot plate) are indeed appropriate to use in these contexts.

These results have interesting implications for how behavioral effects of opioids are studied in mice, including analgesia, muscle rigidity, and locomotion. Alternatives to hind paw–directed reflexive assays for testing opioid analgesia, particularly in a postsurgical pain model, are still needed, but analysis of paw placement during walking may be an interesting area to look for new approaches. Overall, this research will help refine approaches to evaluating opioids in preclinical research, particularly when applied to mouse pain models.

## Disclosures

The authors have no conflict of interest to declare.
